# Approaching the Discriminatory Work Environment as Stressor: The Protective Role of Job Satisfaction on Health

**DOI:** 10.3389/fpsyg.2016.01313

**Published:** 2016-08-30

**Authors:** Donatella Di Marco, Rocio López-Cabrera, Alicia Arenas, Gabriele Giorgi, Giulio Arcangeli, Nicola Mucci

**Affiliations:** ^1^Department of Social Psychology, University of SevilleSeville, Spain; ^2^Department of Psychology, European University of RomeRome, Italy; ^3^Department of Experimental and Clinical Medicine, University of FlorenceFlorence, Italy

**Keywords:** discriminatory work environment, job satisfaction, employees’ health, human resource management, Italian workers, workplace, work-related stress, occupational medicine

## Abstract

Discrimination is a complex phenomenon with adverse consequences at personal and organizational levels. Past studies have demonstrated that workers who are victims of discrimination might show less job satisfaction, less organizational commitment and worse levels of health and productivity. Although most research has focused on the effects of discrimination on victims, less is known about the extent to which discrimination produces consequences on workers who perceive the existence of a discriminatory work environment. The goal of this article is to analyze the consequences of the perception of a discriminatory work environment on employees’ health. The importance of this relationship is studied taking into account the mediating effect of job satisfaction. In order to reach this goal a cross-sectional study was carried out with a sample of 1633 Italian workers (male = 826, female = 764), employed in private and public sectors, and in different hierarchical positions. Results suggest that the perception of a discriminatory work environment is negatively associated with employees’ health. This relationship is partially mediated by job satisfaction (*R*^2^ = 0.17). This study demonstrates that perceiving a discriminatory work environment might have a negative impact on workers’ health. A higher level of job satisfaction might buffer this effect. These findings have several practical implications. On the one hand, Human Resource Managers need to intervene in order to recognize and diminish implicit biases, creating a healthy and inclusive environment (e.g., through training, diversity policies, etc.). On the other hand, promoting job satisfaction (e.g., providing mechanisms of voice) might help workers to preserve their well-being, coping with the negative effects of a discriminatory work environment.

## Introduction

In the last few years, numerous studies about discrimination at work on the basis of gender and ethnic origin have been carried out (Dovidio et al., 2002; Cortina et al., 2013). Recently, researchers have focused their attention on other vulnerable groups, such as disabled people, lesbian, gay, bisexual and transsexual (LGBT) workers, younger/older employees, people who belong to minority religions or beliefs, people who perform menial or strenuous tasks, people who work at unsocial hours, etc. (Hebl et al., 2002; Baldasseroni et al., 2005; Arcangeli and Mucci, 2009; Mucci et al., 2012; Di Marco et al., 2015). These studies shed light on the prejudicial outcomes of discrimination at personal and organizational levels. Although many societies offer legislative tools to employees who have received discriminatory treatment (Goldman et al., 2006), and the prevalence of open discrimination has decreased, data on discrimination in the workplace appears alarming (Goldman et al., 2006; European Commission, 2012, 2015). According to the last Eurobarometer (European Commission, 2015), European citizens reported an increment in their perceptions of discrimination in their countries, including data about discrimination on the bases of ethnic origin, sexual orientation, gender identity, disability, and religion or beliefs, which has been increasing. The same report showed disheartening data about the perceptions of discrimination in Italy. In fact, much more than half of participants believe that discrimination on the basis of ethnic origin, sexual orientation, gender identity, and disability is widespread (73, 73, 71, and 52%, respectively; European Commission, 2015).

Most of the research in this area has focused on the effects of discrimination on victims, while little is known about the extent to which perceiving a discriminatory environment might have consequences for people who are not directly involved in discrimination. Can the witnesses of discriminatory behaviors be affected by such acts? What are the consequences of perceiving the work environment as discriminatory?

The goal of this article is to analyze the consequences of the perception of a discriminatory environment on workers’ health. Moreover, we try to identify the role of an affective and cognitive mediator (job satisfaction) on this relationship.

The article will be structured in the following way: firstly, we will revise past theoretical contributions on discrimination and health; secondly, we will explain why job satisfaction could mediate the relationship between discrimination and health; and finally, we will describe and discuss the results obtained. Theoretical and practical implications will be presented at the end.

### Workplace Discrimination and Health

The term discrimination refers to an unequal treatment people receive for being part of a specific group (Goldman et al., 2006; Triana et al., 2015).

Prejudice – negative attitude against people who belong to a group – and stereotypes – beliefs related to that group – are responsible for this process. In fact, in order to simplify the world, people tend to associate each person with a specific social category which is, in turn, connected to beliefs that change with the historical moment (Dovidio, 2001). For example, beliefs associated with the role of women in societies have changed throughout history. Some decades ago, their role was restricted to the family domain and women who went beyond this sphere were judged negatively by society (Sánchez, 2012). Nowadays, in western countries, open prejudice and negative stereotypes against working women are not judged positively. However, sometimes people do not know that they are bringing prejudice and stereotypes against a specific group at an implicit level. Indeed, a recent study demonstrated that people still maintain traditional gender beliefs (role behaviors, occupations, etc.; Heines et al., 2016). As discrimination is socially undesirable, prejudice, and stereotypes might be stored in an unconscious level, generating implicit bias which, in turn, might shape a discriminatory work environment. Its consequences are equally as prejudicial for people involved as for those experiencing explicit bias (Cortina, 2008; Jones et al., 2016).

Past studies have analyzed the negative consequences of discriminatory behaviors for victims (e.g., poorer health, lower job satisfaction, etc.) and organizations (e.g., monetary losses, higher job rotation, etc.; Cortina et al., 2001; Ensher et al., 2001; Dipboye and Halverson, 2004; Ragins, 2004; Goldman et al., 2006; Wated and Sanchez, 2006; Pascoe and Smart Richman, 2009; Williams and Mohammed, 2009; Di Marco et al., 2015; Trau, 2015; Triana et al., 2015).

In order to understand the effects of perceived discrimination on well-being, past studies have applied the Job Demand-Resources (JD-R) model (Demerouti et al., 2001), which considers that discriminatory behaviors act as stressors (Volpone and Avery, 2013). Job Demand refers to the physical and psychological efforts required by a job which produce cost for the employee at a physical or a psychological level (Demerouti et al., 2001; Volpone and Avery, 2013). High Job Demand might affect employees’ well-being negatively, and at the same time it might provoke disengagement (Volpone and Avery, 2013). On the contrary, Job Resources, as social support and organizational resources, are those aspects which lead to a decrement of Job Demand and its effects (Demerouti et al., 2001). Moreover, Job Resources enrich the employee, letting him or her participate in the decision making process, and increasing the worker’s control over his or her work, etc. (Demerouti et al., 2001). An expression of the presence of Job Resources is a higher level of job satisfaction (Nielsen et al., 2011; Yeh, 2015).

Previous research has considered perceived discrimination as a stressor or a Job Demand, highlighting the negative consequences on victims’ health (Kessler et al., 1999; Pascoe and Smart Richman, 2009; Volpone and Avery, 2013). In this sense, “Minority stress” is the concept used to refer to the consequences experienced by the victims. It is the process by which “stigma, prejudice, and discrimination create a hostile and stressful social environment that causes mental health problems” (Meyer, 2003) for people who belong to stigmatized groups. However, little is known about the effects of perceiving a discriminatory work environment on employees who are not part of a vulnerable group but who witness discrimination. A study about bullying showed that being bystanders of workplace bullying might affect observers’ health negatively (Vartia, 2001; Hoel et al., 2004; Lutgen-Sandvik et al., 2007). Moreover, research carried out with lesbian and gay (LG) professionals (Trau, 2015) demonstrated that a discriminatory climate might be perceived by workers, which in turn might have consequences at personal and organizational levels (Ragins, 2004). Perceiving a discriminatory environment might be considered a stressor, even if people do not experience discrimination directly. In order to overcome the lack of studies about the effect of perceiving a discriminatory environment on employees who do not belong to a vulnerable group, we are going to test the following hypothesis:

*Hypothesis 1:* The perception of a discriminatory work environment will negatively affect employees’ health.

Past studies have demonstrated that certain people are more vulnerable than others even if they have never experienced discrimination, due to historical reasons. Women (Lim et al., 2008) and blue-collar workers (Gil-González et al., 2013; Mariani et al., 2015) might suffer worse consequences on health than men and white-collar workers, respectively, if they perceive a discriminatory environment. Moreover, although previous research about the prevalence of discrimination in public and private sectors appears inconclusive (Byron, 2010; Leasher and Miller, 2012), we believe that the sector might play a significant role into the experience of workplace discrimination. Italian public and private sectors operate following different procedures; for example, during the hiring and firing processes. Generally, the public sector builds the selection process on rigorous procedures (e.g., a public call followed by a selection based on exams), while, in the private sector, selection might depend on the recruiter’s decision, which might be more likely affected by implicit biases. For the same reason, employees who belong to a stigmatized group might perceive their position as more vulnerable if they work in the private sector. For this reason, we believe it is necessary to explore the extent to which employees’ health is affected more severely by the perception of a discriminatory work environment, depending on the sector. For these reasons the following hypotheses state:

*Hypothesis 2*: The effect of a discriminatory work environment on health will be moderated by gender, sector, and job position.
*Hypothesis 2a*: Women will report poorer health than men when they perceive a discriminatory work environment.
*Hypothesis 2b*: People who work in the private sector will report poorer health than people who work in the public sector when they perceive a discriminatory work environment.
*Hypothesis 2c*: Blue-collar workers will report poorer health than white-collar workers and managers when they perceive a discriminatory work environment.

### Job Satisfaction as Mediator

The research about job satisfaction has a long tradition. Many studies have attempted to recognize its affective and cognitive dimensions, trying to identify the best way to measure it (Brief and Weiss, 2002). Job satisfaction has been defined as a positive evaluation that people express after assessing their job at a cognitive and an affective level (Brief, 1998; Brief and Weiss, 2002; Judge and Kammeyer-Mueller, 2012). Judge and Kammeyer-Mueller’s (2012) revision collected some positive consequences of higher levels of job satisfaction. Satisfied employees showed higher job performance (Edwards et al., 2008), more citizenship behaviors (Kurland and Hasson-Gilad, 2015) and fewer thoughts of leaving (Hom and Kinicki, 2001; Baruch et al., 2016). Also, job satisfaction affects employees’ health (Faragher et al., 2005). Given the numerous outcomes of job satisfaction, it is important to identify possible antecedents which have a positive or detrimental effect on employees’ job satisfaction. Past studies have demonstrated that shared time pressure (Silla and Gamero, 2014), job characteristics (e.g., autonomy, skill variety, etc.; Hackman and Oldham, 1976) and social support (Morgeson and Humphrey, 2006; Baruch et al., 2016) might affect employees’ evaluations of their own jobs positively or negatively.

Moreover, previous research has attributed a valuable role to job satisfaction as a mediator variable (Allisey et al., 2014; Silla and Gamero, 2014; Yuan et al., 2014). In a study carried out by Silla and Gamero (2014), job satisfaction played a mediating role in the relationship between shared time pressure and employees’ self-reported health. They considered job satisfaction as an indicator of work adjustment that refers to “employees’ subjective evaluation of the meaning of their work and the view of themselves as functioning members of their organization” (Silla and Gamero, 2014). Job satisfaction and organizational commitment are seen as indicators of work adjustment. Studies framed by the JD-R model found that job satisfaction might be enhanced by organizational resources (Nielsen et al., 2011; Yeh, 2015), whose presence also reduces the negative effects of Job Demand (Bakker and Demerouti, 2007). In line with this, we can hypothesize that the negative effects of the perception of a discriminatory work environment on health might be eliminated by job satisfaction. Indeed, job satisfaction might work as a mediator:

*Hypothesis 3*: Job satisfaction mediates the relationship between the perception of a discriminatory environment and employees’ health.

## Materials and Methods

### Participants

Data were gathered on several Italian organizations from both private and public sectors, using a set of self-reported questionnaires. Even though a total of 1721 responses were collected (1132 from the private sector and 589 from the public sector), 88 incomplete questionnaires were deleted from further analyses, in order to avoid subsequent statistical biases.

The final sample consisted of a total of 1633 employees, 50.6% male and 46.8% female, who were employed in the public (35.5%) and private (64.3%) sectors on three different hierarchical levels: as managers (12.3%), white collars (62%), and blue collars (15.3%).

This study was designed by means of anonymous self-report questionnaires. Employees from Italian private and public sectors were invited to take part in the research. A letter code was assigned to each participant to guarantee their anonymity based on their grandparents’ and mothers’ initials. Three different scales were used in order to obtain all of the required data regarding participants’ perceptions of their work environment, health, and job satisfaction.

#### Discriminatory Environment

Employees’ perceptions of a discriminatory environment were measured using a seven-item subscale of the Stress Questionnaire (SQ) developed by Mucci et al. (2015). The subscale explores the possibility of suffering higher levels of distress by being discriminated against in one’s organization due to race, age, sexual orientation, religion, disabilities, or ideology. Each item was assessed using a 5-point response scale (from 1 = “strongly disagree” to 5 = “strongly agree”). An example of an item is: “People in this organization may be exposed to stress or risks to a greater extent because of their ideology/way of thinking.” Cronbach’s α value for this scale indicates a high reliability (α = 0.72).

#### Health

Employees’ health was measured using an Italian version of the General Health Questionnaire (GHQ; Goldberg, 1972), developed by Fraccaroli et al. (1991). This scale evaluates participants’ perceptions concerning their general health during the last week. A total of 12 items were rated according to a 4-point scale (Less than usual, No more than usual, More than usual, or Much more than usual). However, in order to analyze the obtained data, responses were transformed into a 4-point Likert scale (from 0 to 3) in which a higher score evidences a higher degree of psychological distress; therefore, participants’ final results in this scale may oscillate between a minimum of 0 points and a maximum of 36 points. An example of an item contained in this questionnaire is “You had the impression of not being able to overcome difficulties.” Cronbach’s α for this test was 0.86.

#### Job Satisfaction

Employees’ job satisfaction at work was measured using a five-item subscale obtained from Hartline and Ferrell’s (1996) questionnaire, which analyses this concept in terms of salary/wage, job security, social support, supervisors’ accuracy in decision making processes, and global satisfaction with the job. Items were graded on a 5-point Likert scale basis (from 1 = “Not satisfied at all” to 5 = “Very satisfied”). For instance, one of the statements that participants scored was: “The degree of security that the job offers me.” The internal consistency of this scale was high (Cronbach’s α = 0.70).

#### Sociodemographic Data

Participants also reported gender (male or female), job position (manager, white-collar, or blue-collar), and work sector (public or private).

### Analyses

Descriptive analyses were conducted for each variable involved in the presented model. Additionally, correlations among the main variables were also analyzed. In order to explore the data, one-way ANOVA analyses were conducted, identifying the impact that gender, job position and sector have on participants’ perceptions of discrimination at work, job satisfaction and health. To test our hypotheses, the influence of employees’ perceived discriminatory work environment on health has been analyzed by means of multiple linear regression analyses, considering the moderating effects that gender, job position, and work sector exert on this relationship. In order to conduct this analysis, moderators were transformed into dummy variables and *Z*-scores for each component, avoiding collinearity biases. Then the multiple regressions were calculated.

Finally, simple regression analyses and a mediation analysis based on multiple regression processes were conducted using the PROCESS macro for SPSS (Model 4) developed by Hayes (2013), in order to analyze how job satisfaction influences the relationship between employees’ perceptions of a discriminatory work environment and their health, as well as the ways in which gender, sector, and job position affect the relationship between a discriminatory environment and health.

## Results

Internal consistency results, as well as means, standard deviation, and Pearson correlations among the main variables, are presented in **Table 1**. Regarding the perceived discriminatory work environment (*M* = 2.07, *SD* = 0.70), on average, participants report intermediate levels of this variable. Punctuations of job satisfaction (*M* = 3.50, *SD* = 0.70) reflect a relatively high level of satisfaction at work. Finally, participants report moderated levels of health (*M* = 10.65, *SD* = 5.40). In order to interpret these variable correlations, it must be considered that health presents a reverted score; thus, a higher reported value is worse for this variable. Therefore, those who are discriminated against have worse job satisfaction (*r* = -0.40) and worse health (*r* = 0.20).

**Table 1:** Internal consistencies, means, standard deviations, and correlations among variables.

	*M*	*SD*	1	2	3	4	5	6
1. Gender (1, Men; 2, Women)	1.50	0.50	–					
2. Job position (1, Managers; 2, White collars; 3, Blue collars)	2.03	0.55	-0.13^∗∗^	–				
3. Sector (1, Private; 2, Public)	1.36	0.50	0.16^∗∗^	-0.36^∗∗^	–			
4. Discriminatory work environment	2.07	0.70	-0.10^∗∗^	0.22^∗∗^	-0.01	(0.72)		
5. Job satisfaction	3.50	0.70	-0.02	-0.13^∗∗^	-0.08^∗∗^	-0.32^∗∗^	(0.70)	
6. Health	10.65	5.40	0.06^∗^	-0.03	0.03	0.20^∗∗^	-0.40^∗∗^	(0.86)

*N = 1633; ^∗^*p* < 0.05; ^∗∗^*p* < 0.01. Internal consistencies among of each variable are shown between parentheses.*

Regarding the one-way ANOVA analyses conducted, results suggest that workers’ perceptions of discriminatory work environment, as well as their satisfaction and health, are related to personal and organizational factors such as their gender and job position and the sector to which they belong.

The statistically significant results obtained show that men (*M* = 2.13, *SD* = 0.74) perceive a more discriminatory work environment than women [*M* = 1.99, *SD* = 0.64; *F*(1,1588) = 16.47, *p* = 0.000]. Differences between private (*M* = 2.07, *SD* = 0.71) and public (*M* = 2.06, *SD* = 0.68) sectors regarding the discriminatory work environment are not statistically significant. The results are significant for blue-collar workers [*F*(2,1460) = 38.60, *p* = 0.000], who reported higher scores in those items concerning the discriminatory environment compared to white-collar workers and managers. *Post hoc* comparisons using the Bonferroni test indicate that the mean scores for managers (*M* = 1.83, *SD* = 0.60), white-collar workers (*M* = 2.04; *SD* = 0.70), and blue-collar employees (*M* = 2.38; *SD* = 0.69) are significantly different (*p* < 0.05) regarding the perceived discriminatory environment.

Moreover, there are no statistically significant differences between male and female workers regarding their reported job satisfaction. However, analyses show that job satisfaction is statistically and significantly higher among private company workers (*M* = 3.53, *SD* = 0.71) than among public sector ones [*M* = 3.40, *SD* = 0.67; *F*(1,1631) = 11.53, *p* = 0.001]. Along the same line, job satisfaction is lower among blue-collar workers than among white-collar workers and ultimately managers [*F*(2,1460) = 13.74, *p* = 0.000]. The Bonferroni test results also indicate that there are significant differences (*p* < 0.05) between job positions: managers (*M* = 3.68; *SD* = 0.64), white-collar workers (*M* = 3.48; *SD* = 0.69), and blue-collar workers (*M* = 3.34; *SD* = 0.72).

Concerning participants’ health, the results show that men (*M* = 10.33, *SD* = 5.09) perceive themselves to be healthier than their female coworkers [*M* = 10.95, *SD* = 5.57; *F*(1,1588) = 5.36, *p* = 0.021]. Differences between private and public sectors in relation to workers’ perceptions of health are not significant. Finally, white-collar workers report less health results than managers and blue-collar workers [*F*(2,1460) = 3.56, *p* = 0.030]. *Post hoc* comparisons using the Bonferroni test indicate that the differences between the means for white-collar workers (*M* = 10.95; *SD* = 5.68) and those for blue-collar workers (*M* = 9.97; *SD* = 5.28) with regard to health punctuations are statistically significant (*p* < 0.05); however, that it is not the case for managers (*M* = 10.44; *SD* = 4.27).

Multiple linear regression analyses were conducted to analyze how, on the one hand, a perceived discriminatory work environment influences employees’ health and, on the other hand, how gender, job position, and sector affect that relationship as moderators. The results obtained show that the perception of a discriminatory work environment (β = 0.19, *t* = 7.72; *p* < 0.001) has a statistically significant effect on employees’ health, explaining 3.5% of the variance [*R*^2^ = 0.035; *F*(1,1631) = 59.65; *p* < 0.001]. Therefore, H1 is confirmed; perceiving a discriminatory workplace impairs employees’ health.

In order to test H2, employees’ gender (H2a), sector (H2b), and job position (H2c) were included in the regression analyses as moderators (**Table 2**). Regarding gender, Model 1 explains 4.3% (*R*^2^ = 0.043) of the variance [*F*(2,1587) = 35.30; *p* < 0.001], so discriminatory environment and gender have a statistically significant effect on health as independent variables. However, although Model 2, which explains 4.3% (*R*^2^ = 0.043) of the variance, is statistically significant [*F*(3,1586) = 23.75; *p <* 0.001], the moderation effect of gender is not. In fact, the F change is not statistically significant either, as is shown in **Table 2**. The Durbin-Watson *d* = 1.97, which is between the two critical values of 1.5 < d < 2.5, indicating that there is no first order linear auto-correlation in the multiple linear regression data.

**Table 2:** Change statistics for multiple regression models at values of moderators.

Moderator	Model	*R*^2^	Change statistics			Durbin–Watson
			*R*^2^ change	*F* change	*p* (*F*)	
Gender	1	0.043	0.043	35.30	0.000	1.97
	2	0.043	0.000	0.70	0.405	
Sector	1	0.037	0.037	30.94	0.000	
	2	0.037	0.001	1.15	0.284	1.99
Job position	1	0.045	0.045	34.72	0.000	
(White collars)	2	0.045	0.000	0.054	0.816	1.96
Job position	1	0.050	0.050	38.55	0.000	
(Blue collars)	2	0.051	0.001	0.932	0.334	1.96

Concerning the work sector, Model 1 explains 3.7% (*R*^2^ = 0.037) of the variance [*F*(2,1630) = 30.94; *p* < 0.001], showing that the direct effects of a discriminatory environment and work sector on health are significant. However, Model 2, which explains the same percentage of variance, 3.7% (*R*^2^ = 0.037), indicates that although the model is statistically significant [*F*(3,1629) = 21.01; *p* < 0.001], the work sector has no moderation effect. As is presented on **Table 2**, the F change is not statistically significant. There is no first order linear auto-correlation in the multiple linear regression data, since the Durbin-Watson *d* = 1.99 value is between the two critical values of 1.5 < d < 2.5.

Finally, the job position as moderator has been analyzed based on white-collar workers and blue-collar workers, given the dummy codification of the variables. In terms of white-collar workers, Model 1 explains 4.5% (*R*^2^ = 0.045) of the total variance [*F*(2,1460) = 34.72; *p <* 0.001], so the discriminatory environment and working as a white-collar worker as an independent variable have a statistically significant effect on health. According to Model 2, which explains 4.5% (*R*^2^ = 0.045) of the total variance, it is statistically significant [*F*(3,1459) = 23.15; *p* < 0.001]; however, as is shown in **Table 2**, there is no significant moderation effect of this job position. Indeed, the F change is not statistically significant either. The Durbin-Watson *d* = 1.96, which is between the two critical values of 1.5 < d < 2.5, indicates that there is no first order linear auto-correlation in the multiple linear regression data. Similar results were obtained considering blue-collar workers. Model 1 explains 5% (*R*^2^ = 0.05) of the total variance [*F*(2,1460) = 38.55; *p* < 0.001] and Model 2 explains 5.1% (*R*^2^ = 0.05) of the total variance [*F*(3,1459) = 25.53; *p* < 0.001]. Although Model 2 is statistically significant, the moderation effect of this job position is not. Moreover, the F change value is not significant either. The Durbin-Watson *d* = 1.96, which is between the two critical values of 1.5 < d < 2.5, indicates that there is no first order linear auto-correlation in the multiple linear regression data.

To sum up, the results for gender (β = 0.042, *t* = 0.833; *p* > 0.05), sector (β = 0.055, *t* = 1.071; *p* > 0.05), and job position, white-collar (β = -0.13, *t* = -0.233; *p* > 0.05) and blue-collar (β = 0.067, *t* = 0.966; *p* > 0.05) were not statistically significant when their moderation effects were analyzed separately. Therefore, H2, H2a, H2b, and H2c were not confirmed, and it is concluded that the only factor that influences employees’ health is their perception of discrimination in the workplace.

**FIGURE 1:**
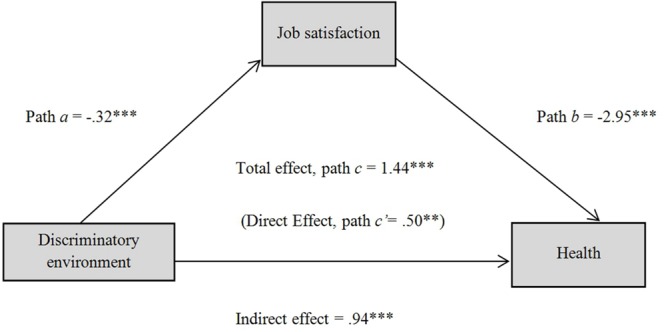
**Mediating effect of job satisfaction on the association between discriminatory environment and health (^∗∗^*p* < 0.01; ^∗∗∗^*p* < 0.001)**.

Mediation analysis (**Table 3**) confirmed the buffering effect of job satisfaction on the relationship between workers’ perceptions of a discriminatory work environment and their health (**Figure 1**). Results revealed that the perception of a discriminatory environment was negatively and significantly related with job satisfaction (path *a, p* < 0.001; *R*^2^ = 0.10) and that job satisfaction was negatively and significantly associated with health (path *b, p* < 0.001; *R*^2^ = 0.17). Additionally, when job satisfaction is included as a mediator, the relationship between discriminatory work environment and health remains marginally significant (total effect, path *c, p* < 0.001; *R*^2^ = 0.03). Also, the resampling procedure (10,000 bootstrap samples) indicates a significant indirect effect, since the confidence interval at 95% does not include the value zero (*k*^2^ = 0.12; bootstrapped 95% CIs of 0.10–0.14; Preacher and Hayes, 2008). This mediation model explains 17% of the employees’ health variance [*F*(2,1630) = 164.70; *p* < 0.001]. Therefore, H3 is partially supported.

**Table 3:** Regression results for mediation.

Variable	*b*	*SE*	*t*	*p*	LLCI	ULCI
**Direct and total effects**					
JS regressed on D (a)	-0.38	0.02	-13.54	0.000	-0.36	-0.27
H regressed on JB, controlling D (b)	-2.95	0.18	-16.13	0.000	-3.31	-2.60
H regressed on D, controlling JB (c)	1.44	0.19	7.72	0.000	1.07	1.81
H regressed on D (c’)	0.50	0.18	2.75	0.006	0.14	0.86
			
	**Value**	***SE***	***z***	***p***		
			
**Indirect effect and significance using normal distribution**					
Sobel	0.94	0.09	10.36	0.000		
			
	***M***	***SE***	**LLCI**	**ULCI**		
			
**Bootstrap results for indirect effect**					
Effect	0.94	0.10	0.76	1.14		

*N = 1633. D, discriminatory environment; JS, job satisfaction; H, health; LL, lower limit; UL, upper limit; CI, confidence interval. Unstandardized regression coefficients are reported. Bootstrap sample size = 10000.*

Further analyses were conducted to go deeply in demographic characteristics that might explain why job satisfaction only has a partial mediation effect on the relationship between a perceived discriminatory environment and health. Based on the ANOVA analyses previously mentioned, regarding job satisfaction, means are significantly different between employees who work in the private and public sectors, as well as between managers, white-collar workers and blue-collar workers; therefore, the hypothesized model was replicated, dividing up the sample according to this classification.

For public sector employees, the results revealed that the perception of a discriminatory environment was negatively and significantly related with job satisfaction (path *a, p* < 0.001; *R*^2^ = 0.073) and that job satisfaction was negatively and significantly associated with health (path *b, p* < 0.001; *R*^2^ = 0.13). Additionally, when job satisfaction is included as a mediator, the relationship between discriminatory work environment and health is not significant (total effect, path *c, p* < 0.001; *R*^2^ = 0.021). Also, the resampling procedure (10,000 bootstrap samples) indicates a significant indirect effect, since the confidence interval at 95% does not include the value zero (*k^2^* = 0.091; bootstrapped 95% CIs of 0.95–0.13; Preacher and Hayes, 2008). This mediation model explains 13% of employees’ health variance [*F*(2,580) = 42.98; *p* < 0.01].

Mediation analysis for the private sector showed that the perception of a discriminatory environment was negatively and significantly related with job satisfaction (path *a, p* < 0.001; *R*^2^ = 0.12) and that job satisfaction was negatively and significantly associated with health (path *b, p* < 0.001; *R*^2^ = 0.20). Additionally, when job satisfaction is included as a mediator, the relationship between discriminatory work environment and health is not significant (total effect, path *c, p* < 0.001; *R*^2^ = 0.045). Also, the resampling procedure (10,000 bootstrap samples) indicates a significant indirect effect, since the confidence interval at 95% does not include the value zero (*k*^2^ = 0.14; bootstrapped 95% CIs of 0.11–0.17; Preacher and Hayes, 2008). This mediation model explains 20% of employees’ health variance [*F*(2,1047) = 122.37; *p* < 0.01].

Regarding job position, for managers the results revealed that the perception of a discriminatory environment was negatively and significantly related with job satisfaction (path *a, p* < 0.001; *R*^2^ = 0.32) and that job satisfaction was negatively and significantly associated with health (path *b, p* < 0.001; *R*^2^ = 0.42). When job satisfaction is included as a mediator, the relationship between discriminatory work environment and health is not significant (total effect, path *c, p* < 0.001; *R*^2^ = 0.08). Also, the resampling procedure (10,000 bootstrap samples) indicates a significant indirect effect, since the confidence interval at 95% does not include the value zero (*k*^2^ = 0.10; bootstrapped 95% CIs of 0.047–0.18; Preacher and Hayes, 2008). This mediation model explains 42% of employees’ health variance [*F*(2,198) = 21.43; *p* < 0.01].

The results revealed that when the hypothesized model was tested for white-collar workers, the perception of a discriminatory environment was negatively and significantly correlated with job satisfaction (path *a, p* < 0.001; *R^2^* = 0.10) and that job satisfaction was negatively and significantly associated with health (path *b, p* < 0.001; *R*^2^ = 0.21). Also, when job satisfaction is included as a mediator, the relationship between discriminatory work environment and health is not significant (total effect, path *c, p* < 0.001; *R*^2^ = 0.036). Also, the resampling procedure (10,000 bootstrap samples) indicates a significant indirect effect, since the confidence interval at 95% does not include the value zero (*k*^2^ = 0.14; bootstrapped 95% CIs of 0.015–0.056; Preacher and Hayes, 2008). This mediation model explains 21% of employees’ health variance [*F*(2,1009) = 133.22; *p* < 0.01].

**FIGURE 2:**
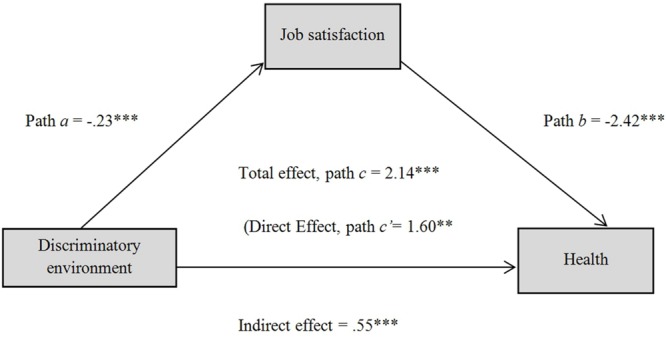
**Mediating effect of job satisfaction on the association between discriminatory environment and health for blue collars (^∗∗^*p* < 0.01; ^∗∗∗^*p* < 0.001)**.

Finally, the mediation analysis results for blue-collar workers are presented in **Figure 2**. They revealed that the perception of a discriminatory environment was negatively and significantly related with job satisfaction (path *a, p* < 0.001; *R*^2^ = 0.046) and that job satisfaction was negatively and significantly associated with health (path *b, p* < 0.001; *R*^2^ = 0.18). Additionally, when job satisfaction is included as a mediator, the relationship between discriminatory work environment and health remains marginally significant (total effect, path *c, p* < 0.001; *R*^2^ = 0.078). Also, the resampling procedure (10,000 bootstrap samples) indicates a significant indirect effect, since the confidence interval at 95% does not include the value zero (*k*^2^ = 0.07; bootstrapped 95% CIs of 0.03–0.081; Preacher and Hayes, 2008). This mediation model explains 18% of employees’ health variance [*F*(2,247) = 27.75; *p* < 0.01]. Therefore, for blue-collar workers, job satisfaction is not a sufficient health protector against a discriminatory environment.

## Discussion

The goal of this study was to understand the consequence of perceiving a discriminatory work environment on employees’ health. Moreover, this research aimed to identify a mediating effect of job satisfaction in the relationship between employees’ perceptions of a discriminatory work environment and their health. With regard to Hypothesis 1, the data confirmed that a relationship exists between employees’ perceptions of a discriminatory work environment and their health. When people consider that their organization is being discriminatory against colleagues who belong to a vulnerable group, their health is affected negatively.

Contrary to expectations, this relationship is not moderated by variables such as gender, job position, or work sector (Hypothesis 2). Our findings demonstrated that the negative effect of a discriminatory work environment affects everybody in the organization, regardless of their belonging to a vulnerable group (e.g., women), regardless of the position they occupy within the organization (e.g., blue-collar workers) and regardless of the work sector. Therefore, Hypotheses 2a, 2b, and 2c were not confirmed.

With regard to Hypothesis 3, our findings showed that the negative relationship between perceiving a discriminatory work environment and employees’ health is partially mediated by job satisfaction. Thus, if employees are satisfied, the impact of perceiving a discriminatory work environment on workers’ health is lower but it still exists. Additional analysis was carried out after dividing up the sample, taking into account work sector and job position. The results demonstrated that job satisfaction almost always mediates totally the relationships between the perceptions of discrimination and workers’ health. However, the mediation is still partial when the model is applied to blue-collar employees alone. For them, job satisfaction is not sufficient in order to eliminate the negative effect of perceiving a discriminatory environment on their health. Therefore, Hypothesis 3 was partially supported.

These findings are in line with studies framed by the JD-R model (Demerouti et al., 2001). According to the JD-R model, workplace discrimination acts as a stressor, as a demand which has negative consequences on both victims and witnesses’ health (Vartia, 2001; Hoel et al., 2004; Lutgen-Sandvik et al., 2007). In line with past studies, which pointed out the effects on health for bystanders of bullying (Hoel et al., 2004), witnessing discrimination might create a climate of fear and people who are bystanders might anticipate the possibility of becoming the next victims of discriminatory acts. Moreover, people who do not support colleagues who have been discriminated against may feel guilty for not intervening (Hoel et al., 2004). Perceiving discriminatory acts does not have an effect on employees’ health alone. Recent studies have demonstrated that negative or rude behaviors might be “contagious”; people who are either victims or bystanders of uncivil acts might enter into a spiral of aggression, behaving in the same manner with other colleagues (Andersson and Pearson, 1999; D’Cruz and Noronha, 2011). The negative effects of discrimination might increase greatly as well as the prejudicial consequences at personal and organizational levels.

This study has several theoretical and practical implications. At a theoretical level, this study extends the knowledge about the role played by job satisfaction as a mediating variable (Silla and Gamero, 2014). Also, our findings demonstrate that a discriminatory workplace is a danger to the whole organization; if it is obvious that it affects vulnerable groups, then it is also true that everybody is a victim of a discriminatory environment. Due to methodological reasons, we cannot talk about “a climate of discrimination,” even though it is reasonable to suppose that it exists (Trau, 2015). Future studies should explore this issue.

This research also has practical implications. Due to the positive effect of job satisfaction on employees’ health, organizations should try to improve this variable. According to the JD-R model (Demerouti et al., 2001; Bakker and Demerouti, 2007), job satisfaction is seen as an indicator of the balance between job demands and organizational resources. Therefore, Human Resource Managers should work to equilibrate such balance, fostering social support, improving autonomy, giving career opportunities, eliminating role ambiguity and role conflict, enriching the task, etc. During this process, it is important to take into account the needs of specific groups (such as blue-collar workers). In order to identify which elements are effective in increasing job satisfaction, mechanisms of voice might be applied (Van Dyne et al., 2003; Verhezen, 2010).

Future studies should explore why job satisfaction loses part of its mediating effect in the case of blue-collar workers. Also, future research should analyze which other factors might be improved in order to protect the health of this work group from the prejudicial consequences of perceiving a discriminatory workplace. Researchers have demonstrated that the perception of justice (Wu et al., 2012) and trust (Chancey et al., 2015) might play an important role within the organization. Future research should investigate whether their presence is effective in eliminating the detrimental role played by perceiving a discriminatory work environment on employees’ health.

However, organizations have to remember that enhancing job satisfaction is not enough; it is important to fight against discrimination at any level. In this line, eliminating implicit biases is the starting point. Educate people about behaviors that transmit implicit discrimination, eliminate stereotypical beliefs about vulnerable groups, engage key organizational actors in activities which combat discriminatory behaviors (Ross, 2014), and create a trusting climate (Capell et al., 2016); all of these are necessary for shaping a safe environment for all employees.

To conclude, some limitations should be pointed out. Firstly, this is a cross-sectional study. Therefore, it is impossible to state robust conclusions about the causality or direction of the mediation (Zapft et al., 1996), given that data were collected at one time. Moreover, data were gathered using a self-reported questionnaire. Another limitation is connected to the number and type of sociodemographic variables collected. Future studies should include variables such as age. Also, other mediating variables might affect the relationship between perceiving a discriminatory work environment and employees’ health (e.g., organizational justice). Finally, we do not have any information about participants’ experience of discrimination as victims. Therefore, being victims of discrimination might have strengthened the relationship between the variables analyzed.

Although many societies have overcome many forms of open discrimination, discriminatory acts are still present at a subtle level. Discrimination does not only generate adverse consequences for people who belong to vulnerable groups. It is a process that involves everybody within the organization. Hence, organizations need to recognize the adverse consequences that these processes might have for victims and bystanders, fostering those aspects that help people to eliminate the negative consequences generated by a discriminatory work environment, such as job satisfaction.

## Author Contributions

The authors contributed to the conception and design of the work; the acquisition, analysis, and interpretation of data for the work. They drafted the work and revised it critically. The authors gave the final approval of the manuscript before the submission. They participated at any step of the research.

## Conflict of Interest Statement

The authors declare that the research was conducted in the absence of any commercial or financial relationships that could be construed as a potential conflict of interest.
